# Correction of Myopic Astigmatism with Topography-Guided Laser In Situ Keratomileusis (TOPOLINK)

**DOI:** 10.3390/healthcare8040477

**Published:** 2020-11-11

**Authors:** Pei-Lun Wu, Chia-Yi Lee, Han-Chih Cheng, Hung-Yu Lin, Li-Ju Lai, Wei-Chi Wu, Hung-Chi Chen

**Affiliations:** 1 Department of Ophthalmology, Chang Gung Memorial Hospital, Chiayi 61301, Taiwan; peilun@cgmh.org.tw (P.-L.W.); lynnlai@cgmh.org.tw (L.-J.L.); 2 Department of Medicine, Chang Gung University College of Medicine, Taoyuan 33302, Taiwan; weichi666@gmail.com; 3 Department of Ophthalmology, Show Chwan Memorial Hospital, Changhua 50093, Taiwan; ao6u.3msn@hotmail.com (C.-Y.L.); anthonyhungyulin@hotmail.com (H.-Y.L.); 4 Department of Ophthalmology, Buddhist Tzu Chi Hospital, Taipei 23142, Taiwan; hanchihcheng@gmail.com; 5 Institute of Medicine, Chung Shan Medical University, Taichung 40201, Taiwan; 6 Department of Optometry, Chung Shan Medical University, Taichung 40201, Taiwan; 7 Department of Ophthalmology, Chang Gung Memorial Hospital, Linkou 33305, Taiwan; 8 Center for Tissue Engineering, Chang Gung Memorial Hospital, Linkou 33305, Taiwan

**Keywords:** refractive error, astigmatism, topography, laser in situ keratomileusis

## Abstract

We aim to assess the feasibility of topography-guided laser in situ keratomileusis (TOPOLINK) for correcting pre-existing and surgical-induced astigmatism. A retrospective, single center cohort study was conducted. Patients with pre-existing irregular myopic astigmatism were recruited into the primary group and those with irregular myopic astigmatism following laser in situ keratomileusis (LASIK) were recruited into the enhancement group. The changes in uncorrected visual acuity (UCVA), best-corrected visual acuity (BCVA), maximum astigmatism, spherical equivalent (SE) and patient satisfaction were recorded. The Chi-square test, Mann–Whitney U test and Generalized Linear Mixed Model were utilized for the analysis in the current study. A total of 18 eyes were studied in the primary group and 14 eyes were examined in the enhancement group. One year postoperatively, the UCVA, BCVA, maximum astigmatism and SE improved significantly in both the primary and the enhancement groups (all *p* < 0.05). The UCVA (*p* = 0.046) and SE (*p* = 0.003) were worse in the primary group preoperatively but became similar in both groups postoperatively, while the BCVA and maximum astigmatism remained identical between groups throughout the study period (all *p* < 0.05). In addition, the rate of high and moderate satisfaction reached 90.0% in the primary and the enhancement groups, without significant differences (*p* = 0.871). In conclusion, the TOPOLINK showed high predictability and will contribute to similar outcomes between primary and postoperative irregular myopic astigmatism concerning visual acuity, refractive status and subject satisfaction.

## 1. Introduction

Astigmatism, first described in the early 1880s, is a refractive condition that leads to the defocusing of images and is frequently encountered by ophthalmologists in clinical practice [1]. Regarding the epidemiology, astigmatism is present in 90% of the human population, and more than 1.0 diopter (D) of astigmatism is present in nearly 25% of the population [2]. The causes of astigmatism include uneven curvature of the cornea and irregularity of the crystalline lens surface, and corneal astigmatism can be categorized into different types if the meridians of maximum and minimum refractive power exist, which is known as regular astigmatism [1]. The etiology of astigmatism is multifactorial and an association between astigmatism and myopia has been found [3,4], and the failure to correct astigmatism in childhood is known to lead to amblyopia [5]. Irregular astigmatism refers to conditions with multiple prominent meridians of refractive power whereby refracted rays have no planes of symmetry, and irregular astigmatism may result from trauma, infection or postoperative status [6]. Irregular astigmatism can also cause loss of best-corrected visual acuity (BCVA), monocular diplopia, ghosting and starburst [7]. Although conventional forms of management, such as glasses, rigid gas permeable contact lenses and toric soft contact lenses, can generally reduce astigmatism, failure to correct astigmatism still occurs in some conditions [8].

Recently, keratorefractive surgery has been developed for the correction of astigmatism, with laser-in-situ keratomileusis (LASIK) and photorefractive keratectomy (PRK) being the most commonly operated corneal refractive surgeries [8]. Despite the fact that LASIK yields a high success rate, postoperative residual or induced astigmatism may limit uncorrected visual acuity (UCVA) and cause glare at night when the conventional technique is used [9]. Customized corneal ablations to treat refractive errors and corneal irregularities using LASIK or PRK, with the assistance of corneal topography, whole-eye wavefront or corneal wavefront techniques, have proven to improve the visual outcome and diminish postoperative complications [10,11]. However, postoperative astigmatism remains a major issue for wavefront-guided LASIK [12].

Several studies showed that topography-based ablations are safe and effective for the treatment of primary myopia and astigmatism compared to wavefront-guided LASIK or small incision lenticule extraction [11,13,14]. Furthermore, corneal topographic examination may be the most useful of the modalities for measuring astigmatism after LASIK, particularly such irregularities as decentration of the ablation and central islands [15]. Besides the above, there is insufficient information regarding topography-guided LASIK (TOPOLINK) and postoperative astigmatism, with a few studies revealing that TOPOLINK can significantly improve the subjective quality of vision and symptoms in patients with irregular corneal astigmatism due to previous laser keratorefractive interventions [16,17]. Moreover, although previous studies have already reported the efficiency of TOPOLINK on both primary and postoperative astigmatism, whether the application of TOPOLINK can yield similar visual acuity and refractive status between the primary and postoperative astigmatisms needs further validation.

Herein, the aim of our study was to assess whether TOPOLINK can correct primary or postoperative irregular astigmatism with similar visual acuity and refraction. Moreover, the alternation of postoperative visual acuity and subjective satisfaction was also discussed.

## 2. Materials and Methods

### 2.1. Ethics Declaration

All the procedures and managements performed in the current study that involved a human patient adhered to the Declaration of Helsinki in 1964 and its late amendment, and the current study was approved by the Institutional Review Board of Chang Gung Memorial Hospital (Project identification code: 201800250B0). Moreover, the Institutional Review Board has waived the requirement of informed consent from patients due to the retrospective nature of this study.

### 2.2. Subject Selection

A retrospective cohort study was conducted in Chang Gung Memorial Hospital, for which the medical records were reviewed. The inclusion criteria were (1) myopia of −1.00 to −7.00 D for the patient without previous refractive surgery, (2) myopia of −1.00 to −4.00 D for the patients with previous LASIK management and (3) presence of irregular myopic astigmatism according to the topography for all patients. The exclusion criteria included (1) previous corneal refractive surgery other than LASIK, (2) the diagnosis of amblyopia, (3) the diagnosis of corneal disorders like dry eye syndrome, corneal scars and keratoconus, (4) the diagnosis of glaucoma, (5) the diagnosis of cataract, (6) the diagnosis of retinal or macular degeneration and (7) maximum astigmatism value more than −3.50 D. The eyes of each patient that met the selection criteria were further divided into two groups: the primary group, which received primary LASIK for primary myopic astigmatism, and the enhancement group, which received enhancement LASIK for residual astigmatism resulting from previous refractive surgery. Then, the data of eyes in both groups were analyzed in a statistical model.

### 2.3. Surgery Technique

All the surgical procedures were performed by one surgeon (Samuel CM Huang). After a 130-mm-thick plate was placed on the eye’s surface, the Moria I microkeratome (Moria SA, Paris, France) was used to cut an 8.5-mm hinged flap. After the cut, suction was released and the corneal flap was then carefully displaced upward; then, ablation was performed using the Schwind excimer laser (Schwind, Kleinostheim, Germany) with a software ablation program based on corneal topography. After the ablation, the back of the flap and the stromal bed were irrigated and the flap was closed and we waited for 5 min to ensure proper adhesion. After surgery, balanced salt solution (Alcon Inc., Fort Worth, TX, USA) and fluorometholone 0.1% (Alcon Inc., Fort Worth, TX, USA) were administered, and the eye was covered with a hard shield for the first night. Topical treatment with eye drops was continued for at least two weeks. Because we selected patients retrospectively, all the participants needed to pay for the surgery, which avoided the possibility of free refractive management leading to overestimated satisfaction.

### 2.4. Main Outcome Measures

All patients were examined preoperatively, three months postoperatively, six months postoperatively and one year postoperatively by one ophthalmologist (SCMH). Both the UCVA and BCVA were tested using projection Snellen chart and presented by a logarithmic scale of minimal angle of resolution (LogMAR). For the residual astigmatism, auto-keratorefractometer (KR-7000, Topcon, Yamagata, Japan) was applied to measure the spherical and cylinder errors which transferred to maximum astigmatism and spherical equivalent (SE) in the analysis. In addition, a short questionnaire was completed three months after surgery by all patients. Patients were asked to rate their satisfaction regarding the result of the surgery as either “highly satisfied”, “moderately satisfied” or “not satisfied”. If participants demanded a re-operation, additional LASIK would be arranged by the same surgeon. To standardize the frequency of refractive surgery, however, the results of the secondary surgery demanded from patients was not included in the following analysis.

### 2.5. Statistical Analysis

All the data in this study were analyzed by using the SPSS 20.0 version (SPSS Inc., Chicago, IL, USA). The descriptive analysis was presented in the form of means ± standard deviations (SD) or frequencies. For basic characteristics, the Chi-square test was used to compare the differences of nominal variables, while the Mann–Whitney U test was applied to analyze the continuous variables. After this, the Generalized Linear Mixed Model was utilized to evaluate the changes in UCVA, BCVA, max astigmatism and SE at different time points (baseline, 3 months, 6 months and one year postoperatively) for both the primary and enhancement groups by incorporating the four parameters into the analysis to eliminate the effects of a possible interaction of these parameters on the surgical outcome. In addition, a comparison between the primary and enhancement groups concerning UCVA, BCVA, max astigmatism and SE was also performed via the same method with Generalized Linear Mixed Model, and the results were demonstrated as bar charts. Furthermore, we created a before–after scatter plot between groups to illustrate the change in each eye rather than just the average value in both groups. For the postoperative satisfaction, the Chi-square test was used again to survey whether the distributions of satisfaction between the two groups had any difference. A *p* value less than 0.05 was regarded as significant, with a confidential interval of 95%. If the *p* value was less than 0.001, the value would be presented as *p* < 0.001.

## 3. Results

### 3.1. Patient Characteristics

A total number of 32 eyes from 20 patients were examined, with 18 eyes from 10 patients assessed in the primary group and another 14 eyes from 10 subjects were assessed in the enhancement group. The statistical power reached 0.79 under the 0.05 alpha value and medium effect size using G*power version 3.1.9.2 (Heinrich-Heine-Universität, Düsseldorf, Germany). There were no differences concerning the age and laterality of operated eyes, but the size of the optic zone and the ablation depth were significantly larger in the primary group (both *p*< 0.05). Besides the above, a female dominance was observed in the enhancement group compared to the primary group (*p* < 0.001). The details of patient demography are shown in Table 1.

### 3.2. Visual Outcomes and Refractive Status in the Primary and Enhancement TOPOLINK

After the follow-up interval of one year, a significant improvement in both mean UCVA (from 1.55 to 0.55, *p* < 0.001) and mean BCVA (from 0.77 to 0.28, *p* < 0.001) was observed after the treatment of TOPOLINK in the primary group. In addition, a reduction in maximum astigmatism (from −0.64 to −0.08, *p* < 0.001) and mean SE (from −5.26 to −0.71, *p* < 0.001), which correlated to the preoperative status, was also confirmed in the primary group (Table 2). In the enhancement group, on the other hand, a similar trend was also revealed, with improvements in mean UCVA (from 1.10 to 0.51, *p* = 0.013), mean BCVA (from 0.55 to 0.25, *p* = 0.047), maximum astigmatism (from −0.75 to −0.13, *p* = 0.021) and mean SE (from −2.50 to −0.43, *p* = 0.033) (Table 3). Comparing the differences between the primary group and the enhancement group, a better UCVA was found in the enhancement group preoperatively (*p* = 0.046). However, there were no significant differences in UCVA between the two groups after the arrangement of TOPOLINK (Figure 1), and the BCVA remained at similar values between the two groups throughout the study period (all *p* > 0.05) (Figure 2). In addition, the maximum astigmatism between the two groups was similar, whether in the preoperative or postoperative category (all *p* > 0.05) (Figure 3). Furthermore, the SE in the primary group was higher than that in the enhancement group before surgery (*p* = 0.003), while the amount of SE showed identical values between the two groups after the surgery (all *p* > 0.05) (Figure 4). The before–after scatter plot that illustrating the changes in all the parameters for each eye in the study population is shown in Figure 5.

### 3.3. Patient Satisfaction

The information regarding subjective satisfaction in both groups is demonstrated in Table 4. In both the primary and enhancement groups, a considerable percentage of patients were satisfied with the visual performance of surgery, with an excellent 90.0 percent of patient feeling that the result could reach the “highly satisfied” or “moderately satisfied” level in both the primary and the enhancement groups. Two patients who were “not satisfied” and “moderately satisfied” in the primary group and one patient who was “not satisfied” in the enhancement group demanded re-operation. The distributions of satisfaction between the two groups showed similar values (*p* = 0.871).

## 4. Discussion

Briefly, the current study demonstrated the feasibility of TOPOLINK for patients with primary irregular astigmatism and postoperative irregular astigmatism via the assistance of a Topography software program. Moreover, TOPOLINK can yield similar UCVA, BCVA, maximum astigmatism and SE outcomes in both primary irregular astigmatism and postoperative irregular astigmatism after one year of follow-up.

Previously, there were different opinions regarding the effectiveness of TOPOLINK for visual improvement. Some reports indicated that customized LASIK guided by topography showed high predictability and efficacy in the correction of myopia and myopic astigmatism ranging from −1.00 to −6.00 D and the improvement of visual acuity [18,19,20]. However, no significant visual recovery was achieved despite the regression of corneal irregularities in the study conducted by Wiesinger-Jendritza et al. [17]. In the current study, both the UCVA and the BCVA were significantly improved after TOPOLINK in both primary astigmatism and postoperatively remnant astigmatism, which showed the positive effect of the tomography-guided ablation technique on visual acuity. Moreover, our study further demonstrated that the final visual acuity outcomes between the two groups were similar. However, in the patients with highly irregular astigmatism (more than 3 D) in our study, the final BCVA could not reach more than 20/40, indicating that the visual improvement of TOPOLINK in high astigmatism individuals is limited. 

For the aspect of refractive correction of TOPOLINK, topography-guided excimer laser ablations including subepithelial keratectomy and PRK have also been used to treat irregular cornea resulting from trauma, corneal surgery, postinfectious disorders and keratoconus [21,22,23,24]. Regarding complicated cases, however, previous studies revealed a significant regression of severe corneal irregularities with decentered and small optic zones due to surgery or trauma in more than 90% of participants, while re-operation was not uncommon for bad visual quality [18,19]. A similar result was found in the study conducted by Alio et al., who found that TOPOLINK was helpful in selected cases where irregular astigmatism showed a defined topography pattern but not in small and extreme irregularities [25]. As a consequence, the use of TOPOLINK in complicated corneal irregularities such as central irregularity or extremely severe astigmatism is not recommended [18,19,25]. In our study, however, the reduction of irregular myopic astigmatism and SE was significant in both the primary group and the enhancement group, and the final values of maximum astigmatism and SE were similar between the two groups, which may indicate the accuracy of TOPOLINK for refractive correction for both primary irregular astigmatism and postoperative astigmatism. Even in high astigmatism patients with an irregular myopic astigmatism above 3 D, the postoperative maximum astigmatism as well as SE can be decreased significantly by a single surgery, which is within our expectations. We used LASIK instead of PRK to avoid the possible influence of epithelial thickness, which tends to be non-uniform in post-surgical corneas, and to minimize the healing change and scarring, especially after repeated surface ablation [18,19,25]. Since most irregular astigmatism following refractive surgery exists at the corneal level [26], it was reasonable to use TOPOLINK to correct gross corneal irregularities in the enhancement group of our study, which contributed to a fair refractive outcome.

Theoretically, customized ablation should be superior to a standard ablation in terms of patient satisfaction since it covers the problem of corneal irregularities or asymmetries that are present with an average amount of 1.25D in the normal human cornea [27]. Moreover, the widespread use of keratorefractive surgery has resulted in a large number of corneal irregularities caused by central islands, small or decentrated optical zones and irregular astigmatism, which cannot be corrected easily via spectacles or refractive surgery [19,25]. The patient satisfaction in our study was high, with only two patients who were “not satisfied” with the visual outcome. For the three patients that received re-operation, two of them were patients who were “not satisfied” with the visual outcome and one patient in the primary group reported being moderately satisfied but requested further improvement. Regarding the two patients who felt unsatisfied with TOPOLINK, one patient belonged to the primary group, whose BCVA was 20/100 and the SE remained at −3.5 D, and another patient was from the enhancement group, whose UCVA and BCVA were around 20/80. The patient felt moderately satisfied but the secondary surgery revealed a BCVA of 20/20, and the patient did not want to wear glasses for the residual SE of around −1.5 D. After the re-arrangement of TOPOLINK, all patients selected the “very satisfied” level after the second TOPOLINK treatment. The result revealed that a second surgery can be recommended for those individuals with poor postoperative satisfaction. 

For topographically-assisted ablation to be developed into an optimal surgical tool in the treatment of corneal irregularities, a few hurdles must be overcome, which involve accurate assessment of the precise preoperative refractive status, limitations in current laser aiming and delivery systems, eye tracking, globe stabilization and the complex mathematics involved in modeling and designing customized laser ablation patterns, which need further improvement for high astigmatism cases [19,20].

There are several limitations in our study. First, the small sample size in the current study might lead to some major statistical bias despite the fact that the case numbers in the current study were similar to some preceding research discussing laser ablation for astigmatism [14,16,17]. Moreover, we did not evaluate changes in contrast sensitivity, which could also be improved after the TOPOLINK procedure [28], and the surface asymmetry index and surface regularity index are also absent. In addition, the current study has a relatively short follow-up time of only one year. Fortunately, the refractive status usually stabilizes one month postoperatively and does not change significantly after the initial experience [23], implying that the length of our follow-up period is sufficient.

## 5. Conclusions

In conclusion, TOPOLINK has shown high predictability and efficacy for the retardation of both primary and postoperative irregular myopic astigmatism, with improvement in visual acuity and reduction of refractive error. Furthermore, the final visual acuity as well as refractive status is similar whether the irregular myopic astigmatism is primary or postoperative. Meanwhile, the subjective satisfaction also reached a high level, with more than 50% of patients considering TOPOLINK as highly satisfactory. Further large-scale trials are needed to evaluate the optimal TOPOLINK pattern for extreme irregular astigmatism.

## Figures and Tables

**Figure 1:**
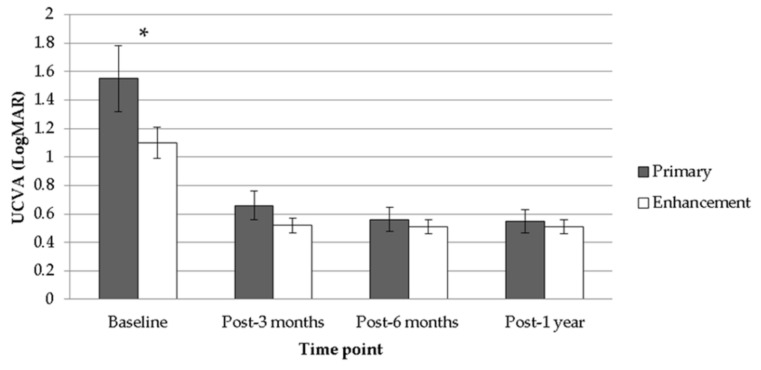
Comparison between the primary group and the enhancement group regarding uncorrected visual acuity. UCVA: uncorrected visual acuity; LogMAR: logarithmic scale of minimal angle of resolution; * denotes significant difference.

**Figure 2:**
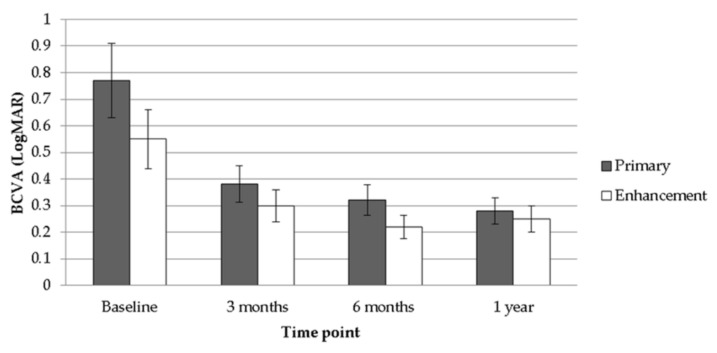
Comparison between the primary group and the enhancement group regarding best-corrected visual acuity. BCVA: best-corrected visual acuity; LogMAR: logarithmic scale of minimal angle of resolution.

**Figure 3:**
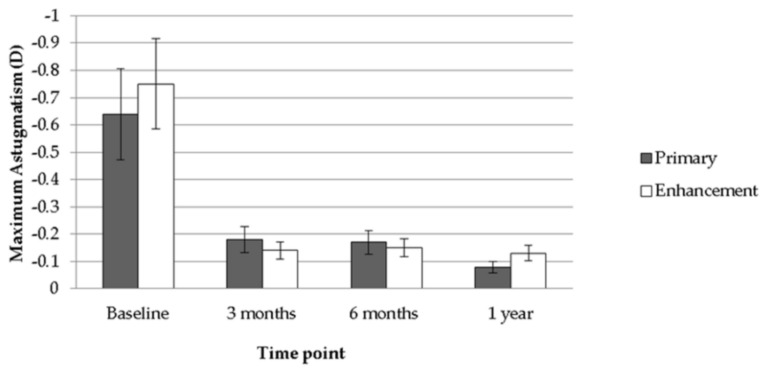
Comparison between the primary group and the enhancement group regarding maximum astigmatism. D: diopter.

**Figure 4:**
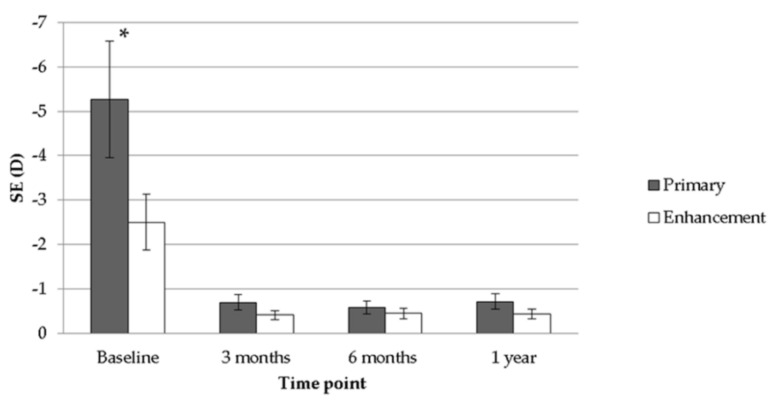
Comparison between the primary group and the enhancement group regarding spherical equivalent. SE: spherical equivalent; D: diopter; * denotes significant difference.

**Figure 5:**
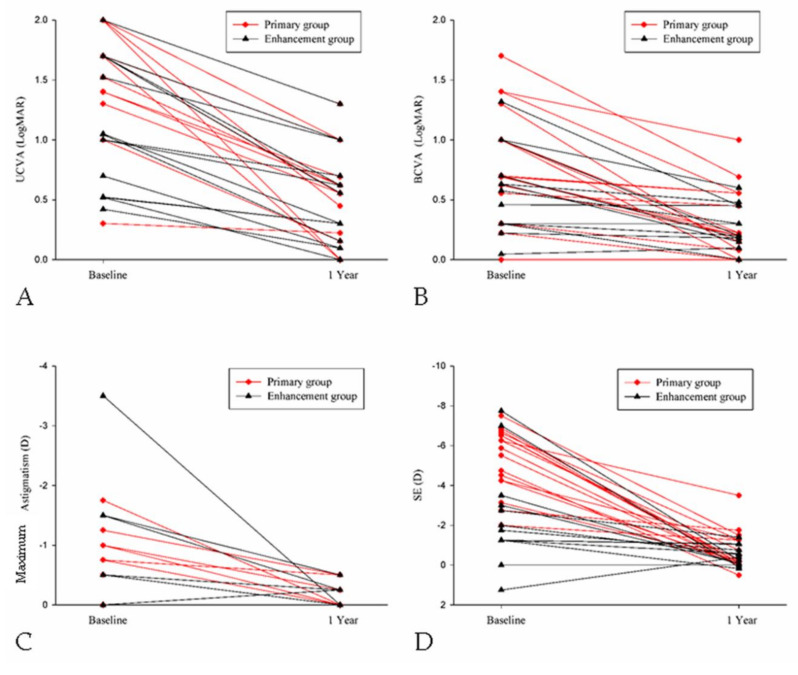
The changes in different parameters after the surgery for each eye in the study population. (**A**) The change in uncorrected visual acuity; (**B**) The change in best-corrected visual acuity; (**C**) The change in maximum astigmatism; (**D**) The change in spherical equivalent. UCVA: uncorrected visual acuity; BCVA: best-corrected visual acuity; SE: spherical equivalent; LogMAR: logarithmic scale of minimal angle of resolution; D: diopter.

**Table 1:** Basic demography.

Demography	Primary Group (*n* = 18)	Enhancement Group (*n* = 14)	*p* Value
Age	27.10 ± 5.55	31.40 ± 7.29	0.545
Sex (male: female)	6:4	8:2	<0.001 *
Laterality (right: left)	9:9	8:6	0.688
Optic zone (mm)	6.39 ± 0.27	6.07 ± 0.28	0.005 *
Ablation depth (µm)	56.06 ± 23.83	43.21 ± 15.24	0.001 *

*n*: number; * denotes significant difference.

**Table 2:** The change in visual acuity and refractive status in the primary group after the topography-guided laser in situ keratomileusis.

Parameters	Baseline	3 Months Postoperative	6 Months Postoperative	1 Year Postoperative	*p* Value
UCVA (LogMAR, mean ± SD)	1.55 ± 0.31 ^A^	0.66 ± 0.27 ^B^	0.56 ± 0.28 ^B^	0.55 ± 0.27 ^B^	<0.001 *
BCVA (LogMAR, mean ± SD)	0.77 ± 0.28 ^A^	0.38 ± 0.25 ^B^	0.32 ± 0.08 ^B^	0.28 ± 0.13 ^B^	<0.001 *
Maximum astigmatism (D, mean ± SD)	−0.64 ± 0.53 ^A^	−0.18 ± 0.32 ^B^	−0.17 ± 0.10 ^B^	−0.08 ± 0.09 ^C^	<0.001 *
SE (D, mean ± SD)	−5.26 ± 1.36 ^A^	−0.69 ± 0.64 ^B^	−0.58 ± 0.39 ^B^	−0.71 ± 0.64 ^B^	<0.001 *

UCVA: uncorrected visual acuity; BCVA: best-corrected visual acuity; SE: spherical equivalent; LogMAR: logarithmic scale of minimal angle of resolution; D: diopter; SD: standard deviation; * denotes significant difference, ^A–C^: intergroup comparison, the same letter represents no significant difference among groups and different letters represent significant difference among groups.

**Table 3:** The change in visual acuity and refractive status in the enhancement group after the topography-guided laser in situ keratomileusis.

Parameters	Baseline	3 Months Postoperative	6 Months Postoperative	1 Year Postoperative	*p* Value
UCVA (LogMAR, mean± SD)	1.10 ± 0.35 ^A^	0.52 ± 0.27 ^B^	0.51 ± 0.15 ^B^	0.51 ± 0.35 ^B^	0.013 *
BCVA (LogMAR, mean± SD)	0.55 ± 0.30 ^A^	0.30 ± 0.27 ^B^	0.22 ± 0.26 ^B^	0.25 ± 0.16 ^B^	0.047 *
Maximum astigmatism (D, mean± SD)	-0.75 ± 0.90 ^A^	-0.14 ± 0.21 ^B^	-0.15 ± 0.08 ^B^	-0.13 ± 0.08 ^B^	0.021 *
SE (D, mean± SD)	-2.50 ± 2.35 ^A^	-0.41 ± 0.43 ^B^	-0.44 ± 0.13 ^B^	-0.43 ± 0.18 ^B^	0.033 *

UCVA: uncorrected visual acuity; BCVA: best-corrected visual acuity; SE: spherical equivalent; LogMAR: logarithmic scale of minimal angle of resolution; D: diopter; SD: standard deviation; * denotes significant difference, ^AB^: intergroup comparison, the same letter represents no significant difference among groups and different letters represent significant difference among groups.

**Table 4:** Patient satisfaction for the topography-guided laser in situ keratomileusis.

Patient Satisfaction	Primary Group (*n* = 10)	Enhancement Group (*n* = 10)	*p* Value
Satisfaction			0.871
Highly satisfied	60.0% (*n* = 6)	70.0% (*n* = 7)	
Moderately satisfied	30.0% (*n* = 3)	20.0% (*n* = 2)	
Not satisfied	10.0% (*n* = 1)	10.0% (*n* = 1)	
Total	100% (*n* = 10)	100% (*n* = 10)	

*n*: number.
